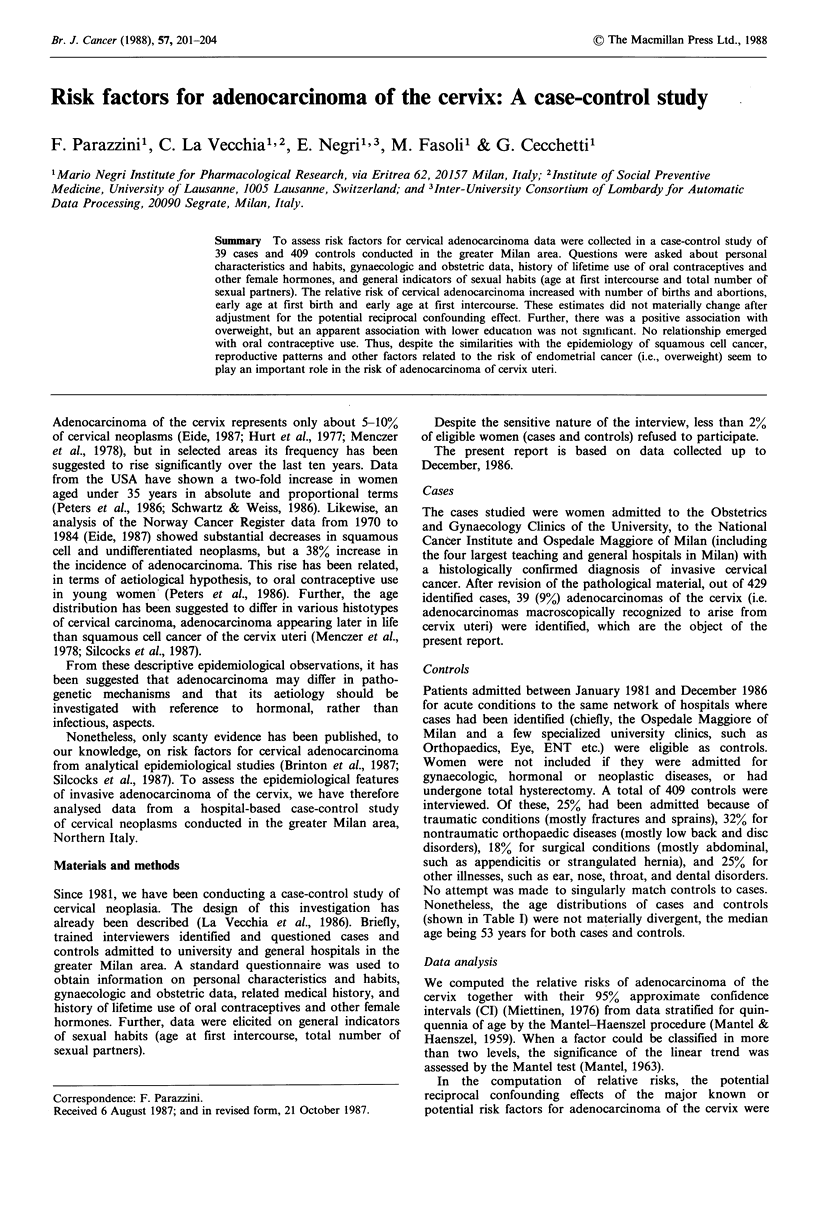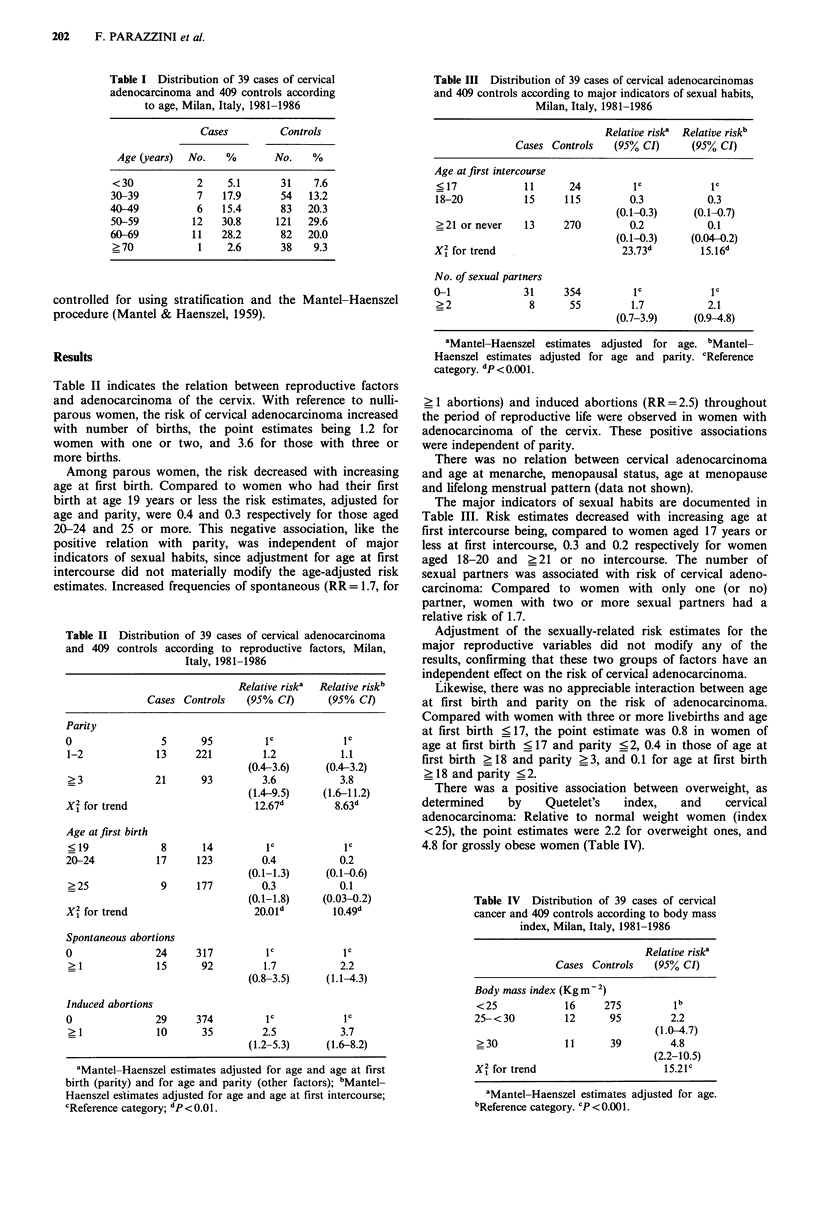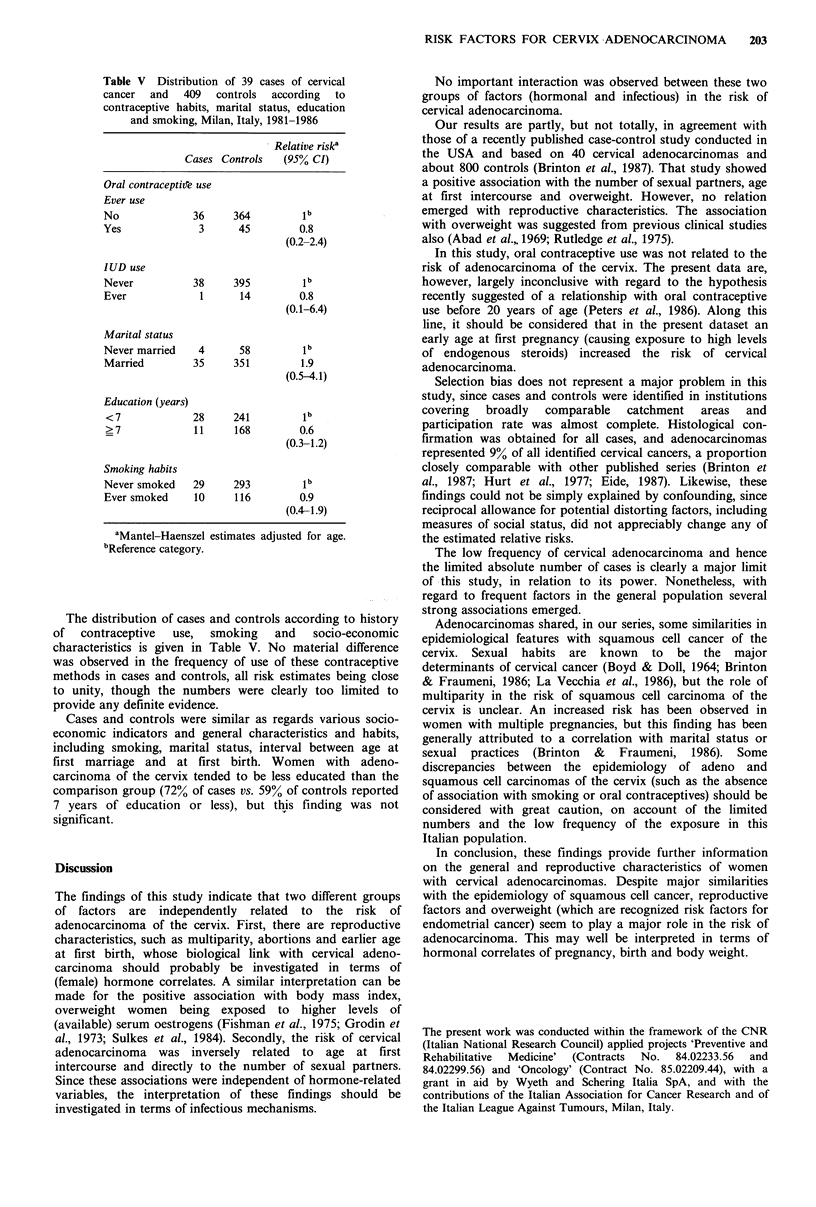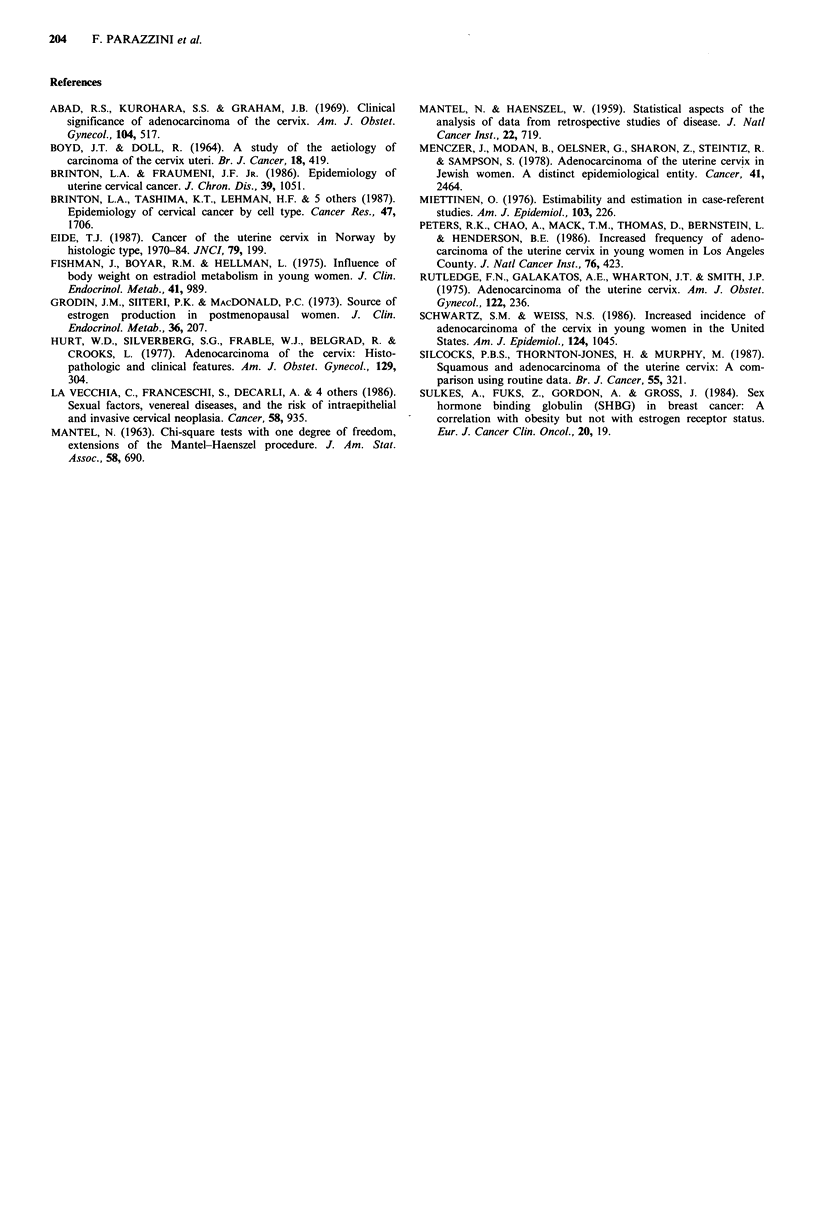# Risk factors for adenocarcinoma of the cervix: a case-control study.

**DOI:** 10.1038/bjc.1988.43

**Published:** 1988-02

**Authors:** F. Parazzini, C. La Vecchia, E. Negri, M. Fasoli, G. Cecchetti

**Affiliations:** Mario Negri Institute for Pharmacological Research, Milan, Italy.

## Abstract

To assess risk factors for cervical adenocarcinoma data were collected in a case-control study of 39 cases and 409 controls conducted in the greater Milan area. Questions were asked about personal characteristics and habits, gynaecologic and obstetric data, history of lifetime use of oral contraceptives and other female hormones, and general indicators of sexual habits (age at first intercourse and total number of sexual partners). The relative risk of cervical adenocarcinoma increased with number of births and abortions, early age at first birth and early age at first intercourse. These estimates did not materially change after adjustment for the potential reciprocal confounding effect. Further, there was a positive association with overweight, but an apparent association with lower education was not significant. No relationship emerged with oral contraceptive use. Thus, despite the similarities with the epidemiology of squamous cell cancer, reproductive patterns and other factors related to the risk of endometrial cancer (i.e., overweight) seem to play an important role in the risk of adenocarcinoma of cervix uteri.


*Br. J. Cancer* (1988), **57**, 201–204 © The Macmillan Press Ltd., 1988

### Risk factors for adenocarcinoma of the cervix: A case-control study

F. Parazzini[1], C. La Vecchia[1,2], E. Negri[1,3], M. Fasoli[1] & G. Cecchetti[1]

[1]*Mario Negri Institute for Pharmacological Research, via Eritrea 62, 20157 Milan, Italy;* [2]*Institute of Social Preventive Medicine, University of Lausanne, 1005 Lausanne, Switzerland; and* [3]*Inter-University Consortium of Lombardy for Automatic Data Processing, 20090 Segrate, Milan, Italy.*

**Summary** To assess risk factors for cervical adenocarcinoma data were collected in a case-control study of 39 cases and 409 controls conducted in the greater Milan area. Questions were asked about personal characteristics and habits, gynaecologic and obstetric data, history of lifetime use of oral contraceptives and other female hormones, and general indicators of sexual habits (age at first intercourse and total number of sexual partners). The relative risk of cervical adenocarcinoma increased with number of births and abortions, early age at first birth and early age at first intercourse. These estimates did not materially change after adjustment for the potential reciprocal confounding effect. Further, there was a positive association with overweight, but an apparent association with lower education was not significant. No relationship emerged with oral contraceptive use. Thus, despite the similarities with the epidemiology of squamous cell cancer, reproductive patterns and other factors related to the risk of endometrial cancer (i.e., overweight) seem to play an important role in the risk of adenocarcinoma of cervix uteri.

Adenocarcinoma of the cervix represents only about 5–10% of cervical neoplasms (Eide, 1987; Hurt *et al.*, 1977; Menczer *et al.*, 1978), but in selected areas its frequency has been suggested to rise significantly over the last ten years. Data from the USA have shown a two-fold increase in women aged under 35 years in absolute and proportional terms (Peters *et al.*, 1986; Schwartz & Weiss, 1986). Likewise, an analysis of the Norway Cancer Register data from 1970 to 1984 (Eide, 1987) showed substantial decreases in squamous cell and undifferentiated neoplasms, but a 38% increase in the incidence of adenocarcinoma. This rise has been related, in terms of aetiological hypothesis, to oral contraceptive use in young women (Peters *et al.*, 1986). Further, the age distribution has been suggested to differ in various histotypes of cervical carcinoma, adenocarcinoma appearing later in life than squamous cell cancer of the cervix uteri (Menczer *et al.*, 1978; Silcocks *et al.*, 1987).

From these descriptive epidemiological observations, it has been suggested that adenocarcinoma may differ in pathogenetic mechanisms and that its aetiology should be investigated with reference to hormonal, rather than infectious, aspects.

Nonetheless, only scanty evidence has been published, to our knowledge, on risk factors for cervical adenocarcinoma from analytical epidemiological studies (Brinton *et al.*, 1987; Silcocks *et al.*, 1987). To assess the epidemiological features of invasive adenocarcinoma of the cervix, we have therefore analysed data from a hospital-based case-control study of cervical neoplasms conducted in the greater Milan area, Northern Italy.

### Materials and methods

Since 1981, we have been conducting a case-control study of cervical neoplasia. The design of this investigation has already been described (La Vecchia *et al.*, 1986). Briefly, trained interviewers identified and questioned cases and controls admitted to university and general hospitals in the greater Milan area. A standard questionnaire was used to obtain information on personal characteristics and habits, gynaecologic and obstetric data, related medical history, and history of lifetime use of oral contraceptives and other female hormones. Further, data were elicited on general indicators of sexual habits (age at first intercourse, total number of sexual partners).

Correspondence: F. Parazzini.
Received 6 August 1987; and in revised form, 21 October 1987.

Despite the sensitive nature of the interview, less than 2% of eligible women (cases and controls) refused to participate.

The present report is based on data collected up to December, 1986.

### *Cases*

The cases studied were women admitted to the Obstetrics and Gynaecology Clinics of the University, to the National Cancer Institute and Ospedale Maggiore of Milan (including the four largest teaching and general hospitals in Milan) with a histologically confirmed diagnosis of invasive cervical cancer. After revision of the pathological material, out of 429 identified cases, 39 (9%) adenocarcinomas of the cervix (i.e. adenocarcinomas macroscopically recognized to arise from cervix uteri) were identified, which are the object of the present report.

### *Controls*

Patients admitted between January 1981 and December 1986 for acute conditions to the same network of hospitals where cases had been identified (chiefly, the Ospedale Maggiore of Milan and a few specialized university clinics, such as Orthopaedics, Eye, ENT etc.) were eligible as controls. Women were not included if they were admitted for gynaecologic, hormonal or neoplastic diseases, or had undergone total hysterectomy. A total of 409 controls were interviewed. Of these, 25% had been admitted because of traumatic conditions (mostly fractures and sprains), 32% for nontraumatic orthopaedic diseases (mostly low back and disc disorders), 18% for surgical conditions (mostly abdominal, such as appendicitis or strangulated hernia), and 25% for other illnesses, such as ear, nose, throat, and dental disorders. No attempt was made to singularly match controls to cases. Nonetheless, the age distributions of cases and controls (shown in Table I) were not materially divergent, the median age being 53 years for both cases and controls.

### *Data analysis*

We computed the relative risks of adenocarcinoma of the cervix together with their 95% approximate confidence intervals (CI) (Miettinen, 1976) from data stratified for quinquennia of age by the Mantel–Haenszel procedure (Mantel & Haenszel, 1959). When a factor could be classified in more than two levels, the significance of the linear trend was assessed by the Mantel test (Mantel, 1963).

In the computation of relative risks, the potential reciprocal confounding effects of the major known or potential risk factors for adenocarcinoma of the cervix were

**Table I** Distribution of 39 cases of cervical adenocarcinoma and 409 controls according to age, Milan, Italy, 1981–1986

| | *Cases* | | *Controls* | |
|---|---|---|---|---|
| *Age (years)* | *No.* | % | *No.* | % |
| <30 | 2 | 5.1 | 31 | 7.6 |
| 30–39 | 7 | 17.9 | 54 | 13.2 |
| 40–49 | 6 | 15.4 | 83 | 20.3 |
| 50–59 | 12 | 30.8 | 121 | 29.6 |
| 60–69 | 11 | 28.2 | 82 | 20.0 |
| ≧70 | 1 | 2.6 | 38 | 9.3 |

controlled for using stratification and the Mantel–Haenszel procedure (Mantel & Haenszel, 1959).

### Results

Table II indicates the relation between reproductive factors and adenocarcinoma of the cervix. With reference to nulliparous women, the risk of cervical adenocarcinoma increased with number of births, the point estimates being 1.2 for women with one or two, and 3.6 for those with three or more births.

Among parous women, the risk decreased with increasing age at first birth. Compared to women who had their first birth at age 19 years or less the risk estimates, adjusted for age and parity, were 0.4 and 0.3 respectively for those aged 20–24 and 25 or more. This negative association, like the positive relation with parity, was independent of major indicators of sexual habits, since adjustment for age at first intercourse did not materially modify the age-adjusted risk estimates. Increased frequencies of spontaneous (RR = 1.7, for ≧1 abortions) and induced abortions (RR = 2.5) throughout the period of reproductive life were observed in women with adenocarcinoma of the cervix. These positive associations were independent of parity.

**Table II** Distribution of 39 cases of cervical adenocarcinoma and 409 controls according to reproductive factors, Milan, Italy, 1981–1986

| | Cases | Controls | *Relative risk*[a] *(95% CI)* | *Relative risk*[b] *(95% CI)* |
|---|---|---|---|---|
| *Parity* | | | | |
| 0 | 5 | 95 | 1[c] | 1[c] |
| 1–2 | 13 | 221 | 1.2 (0.4–3.6) | 1.1 (0.4–3.2) |
| ≧3 | 21 | 93 | 3.6 (1.4–9.5) | 3.8 (1.6–11.2) |
| $X_1^2$ for trend | | | 12.67[d] | 8.63[d] |
| *Age at first birth* | | | | |
| ≦19 | 8 | 14 | 1[c] | 1[c] |
| 20–24 | 17 | 123 | 0.4 (0.1–1.3) | 0.2 (0.1–0.6) |
| ≧25 | 9 | 177 | 0.3 (0.1–1.8) | 0.1 (0.03–0.2) |
| $X_1^2$ for trend | | | 20.01[d] | 10.49[d] |
| *Spontaneous abortions* | | | | |
| 0 | 24 | 317 | 1[c] | 1[c] |
| ≧1 | 15 | 92 | 1.7 (0.8–3.5) | 2.2 (1.1–4.3) |
| *Induced abortions* | | | | |
| 0 | 29 | 374 | 1[c] | 1[c] |
| ≧1 | 10 | 35 | 2.5 (1.2–5.3) | 3.7 (1.6–8.2) |

[a]Mantel–Haenszel estimates adjusted for age and age at first birth (parity) and for age and parity (other factors); [b]Mantel–Haenszel estimates adjusted for age and age at first intercourse; [c]Reference category; [d]$P<0.01$.

**Table III** Distribution of 39 cases of cervical adenocarcinomas and 409 controls according to major indicators of sexual habits, Milan, Italy, 1981–1986

| | Cases | Controls | *Relative risk*[a] *(95% CI)* | *Relative risk*[b] *(95% CI)* |
|---|---|---|---|---|
| *Age at first intercourse* | | | | |
| ≦17 | 11 | 24 | 1[c] | 1[c] |
| 18–20 | 15 | 115 | 0.3 (0.1–0.3) | 0.3 (0.1–0.7) |
| ≧21 or never | 13 | 270 | 0.2 (0.1–0.3) | 0.1 (0.04–0.2) |
| $X_1^2$ for trend | | | 23.73[d] | 15.16[d] |
| *No. of sexual partners* | | | | |
| 0–1 | 31 | 354 | 1[c] | 1[c] |
| ≧2 | 8 | 55 | 1.7 (0.7–3.9) | 2.1 (0.9–4.8) |

[a]Mantel–Haenszel estimates adjusted for age. [b]Mantel–Haenszel estimates adjusted for age and parity. [c]Reference category. [d]$P<0.001$.

There was no relation between cervical adenocarcinoma and age at menarche, menopausal status, age at menopause and lifelong menstrual pattern (data not shown).

The major indicators of sexual habits are documented in Table III. Risk estimates decreased with increasing age at first intercourse being, compared to women aged 17 years or less at first intercourse, 0.3 and 0.2 respectively for women aged 18–20 and ≧21 or no intercourse. The number of sexual partners was associated with risk of cervical adenocarcinoma: Compared to women with only one (or no) partner, women with two or more sexual partners had a relative risk of 1.7.

Adjustment of the sexually-related risk estimates for the major reproductive variables did not modify any of the results, confirming that these two groups of factors have an independent effect on the risk of cervical adenocarcinoma.

Likewise, there was no appreciable interaction between age at first birth and parity on the risk of adenocarcinoma. Compared with women with three or more livebirths and age at first birth ≦17, the point estimate was 0.8 in women of age at first birth ≦17 and parity ≦2, 0.4 in those of age at first birth ≧18 and parity ≧3, and 0.1 for age at first birth ≧18 and parity ≦2.

There was a positive association between overweight, as determined by Quetelet's index, and cervical adenocarcinoma: Relative to normal weight women (index <25), the point estimates were 2.2 for overweight ones, and 4.8 for grossly obese women (Table IV).

**Table IV** Distribution of 39 cases of cervical cancer and 409 controls according to body mass index, Milan, Italy, 1981–1986

| | Cases | Controls | *Relative risk*[a] *(95% CI)* |
|---|---|---|---|
| *Body mass index* ($Kg\,m^{-2}$) | | | |
| <25 | 16 | 275 | 1[b] |
| 25–<30 | 12 | 95 | 2.2 (1.0–4.7) |
| ≧30 | 11 | 39 | 4.8 (2.2–10.5) |
| $X_1^2$ for trend | | | 15.21[c] |

[a]Mantel–Haenszel estimates adjusted for age. [b]Reference category. [c]$P<0.001$.

**Table V** Distribution of 39 cases of cervical cancer and 409 controls according to contraceptive habits, marital status, education and smoking, Milan, Italy, 1981–1986

| | *Cases* | *Controls* | *Relative risk*[a] *(95% CI)* |
|---|---|---|---|
| *Oral contraceptive use* | | | |
| *Ever use* | | | |
| No | 36 | 364 | 1[b] |
| Yes | 3 | 45 | 0.8 (0.2–2.4) |
| *IUD use* | | | |
| Never | 38 | 395 | 1[b] |
| Ever | 1 | 14 | 0.8 (0.1–6.4) |
| *Marital status* | | | |
| Never married | 4 | 58 | 1[b] |
| Married | 35 | 351 | 1.9 (0.5–4.1) |
| *Education (years)* | | | |
| <7 | 28 | 241 | 1[b] |
| ≧7 | 11 | 168 | 0.6 (0.3–1.2) |
| *Smoking habits* | | | |
| Never smoked | 29 | 293 | 1[b] |
| Ever smoked | 10 | 116 | 0.9 (0.4–1.9) |

[a]Mantel–Haenszel estimates adjusted for age.
[b]Reference category.

The distribution of cases and controls according to history of contraceptive use, smoking and socio-economic characteristics is given in Table V. No material difference was observed in the frequency of use of these contraceptive methods in cases and controls, all risk estimates being close to unity, though the numbers were clearly too limited to provide any definite evidence.

Cases and controls were similar as regards various socio-economic indicators and general characteristics and habits, including smoking, marital status, interval between age at first marriage and at first birth. Women with adenocarcinoma of the cervix tended to be less educated than the comparison group (72% of cases *vs.* 59% of controls reported 7 years of education or less), but this finding was not significant.

### Discussion

The findings of this study indicate that two different groups of factors are independently related to the risk of adenocarcinoma of the cervix. First, there are reproductive characteristics, such as multiparity, abortions and earlier age at first birth, whose biological link with cervical adenocarcinoma should probably be investigated in terms of (female) hormone correlates. A similar interpretation can be made for the positive association with body mass index, overweight women being exposed to higher levels of (available) serum oestrogens (Fishman *et al.*, 1975; Grodin *et al.*, 1973; Sulkes *et al.*, 1984). Secondly, the risk of cervical adenocarcinoma was inversely related to age at first intercourse and directly to the number of sexual partners. Since these associations were independent of hormone-related variables, the interpretation of these findings should be investigated in terms of infectious mechanisms.

No important interaction was observed between these two groups of factors (hormonal and infectious) in the risk of cervical adenocarcinoma.

Our results are partly, but not totally, in agreement with those of a recently published case-control study conducted in the USA and based on 40 cervical adenocarcinomas and about 800 controls (Brinton *et al.*, 1987). That study showed a positive association with the number of sexual partners, age at first intercourse and overweight. However, no relation emerged with reproductive characteristics. The association with overweight was suggested from previous clinical studies also (Abad *et al.*, 1969; Rutledge *et al.*, 1975).

In this study, oral contraceptive use was not related to the risk of adenocarcinoma of the cervix. The present data are, however, largely inconclusive with regard to the hypothesis recently suggested of a relationship with oral contraceptive use before 20 years of age (Peters *et al.*, 1986). Along this line, it should be considered that in the present dataset an early age at first pregnancy (causing exposure to high levels of endogenous steroids) increased the risk of cervical adenocarcinoma.

Selection bias does not represent a major problem in this study, since cases and controls were identified in institutions covering broadly comparable catchment areas and participation rate was almost complete. Histological confirmation was obtained for all cases, and adenocarcinomas represented 9% of all identified cervical cancers, a proportion closely comparable with other published series (Brinton *et al.*, 1987; Hurt *et al.*, 1977; Eide, 1987). Likewise, these findings could not be simply explained by confounding, since reciprocal allowance for potential distorting factors, including measures of social status, did not appreciably change any of the estimated relative risks.

The low frequency of cervical adenocarcinoma and hence the limited absolute number of cases is clearly a major limit of this study, in relation to its power. Nonetheless, with regard to frequent factors in the general population several strong associations emerged.

Adenocarcinomas shared, in our series, some similarities in epidemiological features with squamous cell cancer of the cervix. Sexual habits are known to be the major determinants of cervical cancer (Boyd & Doll, 1964; Brinton & Fraumeni, 1986; La Vecchia *et al.*, 1986), but the role of multiparity in the risk of squamous cell carcinoma of the cervix is unclear. An increased risk has been observed in women with multiple pregnancies, but this finding has been generally attributed to a correlation with marital status or sexual practices (Brinton & Fraumeni, 1986). Some discrepancies between the epidemiology of adeno and squamous cell carcinomas of the cervix (such as the absence of association with smoking or oral contraceptives) should be considered with great caution, on account of the limited numbers and the low frequency of the exposure in this Italian population.

In conclusion, these findings provide further information on the general and reproductive characteristics of women with cervical adenocarcinomas. Despite major similarities with the epidemiology of squamous cell cancer, reproductive factors and overweight (which are recognized risk factors for endometrial cancer) seem to play a major role in the risk of adenocarcinoma. This may well be interpreted in terms of hormonal correlates of pregnancy, birth and body weight.

The present work was conducted within the framework of the CNR (Italian National Research Council) applied projects 'Preventive and Rehabilitative Medicine' (Contracts No. 84.02233.56 and 84.02299.56) and 'Oncology' (Contract No. 85.02209.44), with a grant in aid by Wyeth and Schering Italia SpA, and with the contributions of the Italian Association for Cancer Research and of the Italian League Against Tumours, Milan, Italy.

### References

ABAD, R.S., KUROHARA, S.S. & GRAHAM, J.B. (1969). Clinical significance of adenocarcinoma of the cervix. *Am. J. Obstet. Gynecol.*, **104**, 517.

BOYD, J.T. & DOLL, R. (1964). A study of the aetiology of carcinoma of the cervix uteri. *Br. J. Cancer*, **18**, 419.

BRINTON, L.A. & FRAUMENI, J.F. JR. (1986). Epidemiology of uterine cervical cancer. *J. Chron. Dis.*, **39**, 1051.

BRINTON, L.A., TASHIMA, K.T., LEHMAN, H.F. & 5 others (1987). Epidemiology of cervical cancer by cell type. *Cancer Res.*, **47**, 1706.

EIDE, T.J. (1987). Cancer of the uterine cervix in Norway by histologic type, 1970–84. *JNCI*, **79**, 199.

FISHMAN, J., BOYAR, R.M. & HELLMAN, L. (1975). Influence of body weight on estradiol metabolism in young women. *J. Clin. Endocrinol. Metab.*, **41**, 989.

GRODIN, J.M., SIITERI, P.K. & MACDONALD, P.C. (1973). Source of estrogen production in postmenopausal women. *J. Clin. Endocrinol. Metab.*, **36**, 207.

HURT, W.D., SILVERBERG, S.G., FRABLE, W.J., BELGRAD, R. & CROOKS, L. (1977). Adenocarcinoma of the cervix: Histopathologic and clinical features. *Am. J. Obstet. Gynecol.*, **129**, 304.

LA VECCHIA, C., FRANCESCHI, S., DECARLI, A. & 4 others (1986). Sexual factors, venereal diseases, and the risk of intraepithelial and invasive cervical neoplasia. *Cancer*, **58**, 935.

MANTEL, N. (1963). Chi-square tests with one degree of freedom, extensions of the Mantel–Haenszel procedure. *J. Am. Stat. Assoc.*, **58**, 690.

MANTEL, N. & HAENSZEL, W. (1959). Statistical aspects of the analysis of data from retrospective studies of disease. *J. Natl Cancer Inst.*, **22**, 719.

MENCZER, J., MODAN, B., OELSNER, G., SHARON, Z., STEINTIZ, R. & SAMPSON, S. (1978). Adenocarcinoma of the uterine cervix in Jewish women. A distinct epidemiological entity. *Cancer*, **41**, 2464.

MIETTINEN, O. (1976). Estimability and estimation in case-referent studies. *Am. J. Epidemiol.*, **103**, 226.

PETERS, R.K., CHAO, A., MACK, T.M., THOMAS, D., BERNSTEIN, L. & HENDERSON, B.E. (1986). Increased frequency of adenocarcinoma of the uterine cervix in young women in Los Angeles County. *J. Natl Cancer Inst.*, **76**, 423.

RUTLEDGE, F.N., GALAKATOS, A.E., WHARTON, J.T. & SMITH, J.P. (1975). Adenocarcinoma of the uterine cervix. *Am. J. Obstet. Gynecol.*, **122**, 236.

SCHWARTZ, S.M. & WEISS, N.S. (1986). Increased incidence of adenocarcinoma of the cervix in young women in the United States. *Am. J. Epidemiol.*, **124**, 1045.

SILCOCKS, P.B.S., THORNTON-JONES, H. & MURPHY, M. (1987). Squamous and adenocarcinoma of the uterine cervix: A comparison using routine data. *Br. J. Cancer*, **55**, 321.

SULKES, A., FUKS, Z., GORDON, A. & GROSS, J. (1984). Sex hormone binding globulin (SHBG) in breast cancer: A correlation with obesity but not with estrogen receptor status. *Eur. J. Cancer Clin. Oncol.*, **20**, 19.